# The Effect of Hope Therapy on the Management of Hemodialysis Outcomes: A Review Article

**DOI:** 10.7759/cureus.54104

**Published:** 2024-02-13

**Authors:** Nazanin H Bayan, Maryam J Farahani, Negin Sedaghat, Shima Mehrabi, Farzaneh Ramezani

**Affiliations:** 1 Department of Psychology, Islamic Azad University, Roudehen Branch, Tehran, IRN; 2 Department of Psychology, Islamic Azad University,Sari Branch, Tehran, IRN; 3 Department of Psychology, Islamic Azad University, Urmia Branch, Urmia, IRN; 4 Department of Psychology, Islamic Azad University, Kermanshah Branch, Kermanshah, IRN; 5 Department of Pathology, Mashhad University of Medical Sciences, Mashhad, IRN

**Keywords:** chronic renal failure, heath-related quality of life, stress, anxiety, hemodialysis, hope therapy

## Abstract

Hemodialysis can have specific adverse effects, so it's critical to minimize them by employing non-pharmacological techniques. This review's primary goal was to assess how hope therapy affected the treatment of hemodialysis patients. This review was conducted by analyzing the results of previous studies published between 1996 and 2023. We chose sixteen studies in consideration of the inclusion and exclusion criteria and by employing Medical Subject Headings (MeSH) terms to the literature discussed in international databases. The findings of the current study revealed that hope therapy can significantly reduce anxiety, stress, and depression and also considerably increase happiness, quality of life, and adherence to treatment in hemodialysis patients. In addition, effective interventions for improving hope in hemodialysis patients included spiritual counseling, spiritual therapy, stress management training, intervention based on disease perception, positive thinking training, and other similar methods. Based on the findings, we concluded that the caregivers of hemodialysis patients and their families must use other non-pharmacological methods, especially hope therapy, to reduce the adverse outcomes of hemodialysis.

## Introduction and background

Chronic renal failure (CRF) is one of the most significant global health concerns, with an estimated prevalence of 242 cases per one million individuals [1]. Hemodialysis is a practical therapeutic approach for chronic renal failure. However, hemodialysis can increase life expectancy in these patients; it has many physical and psychological problems [2], reduces the quality of life, causes frequent hospitalizations, and increases the costs of treatment and health care [3]. Anxiety, depression, and, in more severe manifestations, hopelessness are the most critical psychological issues among hemodialysis patients [4]. Psychological disorders cause activity intolerance, destroy a person's independence in performing routine life activities, and make the person dependent on self-care [5]. The main effects of this dependency are limitations in social, familial, and professional responsibilities, which affect mental health and lead to depression and hopelessness in hemodialysis patients [6]

Hope includes the components of willpower, thinking power, and planning to reach the goal and recognizing obstacles [7]. It is also recognized as a significant aspect of one's actions and holds the potential to impact feelings of grief and uncertainty positively. Furthermore, hope serves as a potent instrument in combating incapacity caused by a multitude of disorders [8]. Strengthening hope increases awareness, mental health, and identifying solutions to problems [9]. A high level of hope among hemodialysis patients reduces their stress, worry, and sadness and increases their quality of life. Therefore, it seems necessary to carry out interventions to improve and strengthen hope, especially in chronic patients [10].

Dialysis patients undergoing hemodialysis must take numerous medications, which contributes to their hesitancy to take further drugs. Consequently, it appears that medication interventions are required to reduce psychological issues [11]. According to a recent study, the most significant non-drug therapy for hemodialysis patients to feel more hopeful is stress management training [9, 12]. So far, a diverse array of data has been generated in the past to investigate the effectiveness of "hope therapy" in managing outcomes of hemodialysis [11-26]. The objective of the current study was to evaluate and synthesize, as a review study, to assess how hope therapy affected the treatment of hemodialysis patients.

## Review

The current investigation commenced as a comprehensive survey of the literature. To search for previous studies from Medical Subject Headings (MeSH) terms including "chronic renal failure", "hemodialysis patients", "psychological interventions", "psychological distress", "chronic renal diseases", "self-efficacy", "self-management", "non-pharmacological methods", "group hope therapy", "spirituality", "treatment regimens", "drug compliance", "kidney transplantation", "spiritual counseling", "spiritual care", "quality of life", "undergoing hemodialysis", "anxiety", "depression", "coping strategies", "stress management", and "treatment adherence" were used. A total of 53 articles were obtained during the initial phase of the search, covering the period from 1996 to 2023. After applying inclusion and exclusion criteria, we selected 16 studies. The results of the selected studies were then tabulated and arranged based on the authors' names, the year the study was conducted, the sample size, and noteworthy findings.

The results of the studies showed that non-pharmacological methods to create motivation, especially the "hope therapy" method, will reduce anxiety and increase adherence to treatment in hemodialysis patients (Table 1). 

**Table 1:** The key findings of previous studies about the effect of the hope therapy method on the management of hemodialysis outcomes

Author and study year	Sample size	Key findings
Rahimipour et al. (2015) [11]	50	Hemodialysis patients who underwent treatment with the Hope therapy method experienced significant reductions in their mean scores for sadness, anxiety, and tension.
Gerogianni et al. (2019) [12]	-	Hemodialysis patients who participate in spirituality and religiosity, social support, and remote nursing programs experience few symptoms of anxiety and depression and an increased sense of hope.
Ahmadifajr et al. (2023) [13]	40	Researchers reported significant treatment adherents and low levels of anxiety among hemodialysis patients receiving therapy in groups.
Ottaviani et al. (2014) [14]	127	Spirituality and faith have a strong correlation among hemodialysis patients. A stronger correlation exists between an individual's religious beliefs and their level of hope, influencing their degree of optimism regarding recovery, adjustment to their disease, and ongoing medical care.
Cha and Han (2014) [15]	100	The findings of this study revealed that there was a significant influence of occupation, income, and religion on levels of hope among hemodialysis patients. Moreover, the results of this study suggest that hope acts as a protective factor for individuals undergoing hemodialysis, underscoring the necessity for a proficient treatment strategy, specifically for adolescent and adult patients.
Farnia et al. (2016) [16]	48	After the hope therapy intervention, hemodialysis patients reported significantly greater overall satisfaction.
Omranifard et al. (2017) [17]	85	The mean anxiety score did not differ significantly between the two patient groups (those who followed their treatment regimen and those who did not; p=0.210). Conversely, individuals who strictly followed the prescribed medication exhibited a significantly greater mean level of depression (p=0.043). Based on the findings of this research, it is evident that the level of depression among hemodialysis patients has an impact on their adherence to treatment. Consequently, curing depression seems to warrant increased emphasis and attention.
Afazel et al. (2013) [18]	90	The intervention group of hemodialysis patients exhibited a substantially higher average level of hope after spiritual counseling as compared to the control group.
Oshvandi et al. (2020) [19]	60	There was a statistically significant difference in the mean scores of hope between the experimental group and the control group among hemodialysis patients who received spiritual care.
Saki et al. (2022) [20]	84	In comparison to the control group, the intervention group exhibited a statistically significant decrease in the mean scores of death anxiety levels and a considerable increase in the mean scores of hope levels.
Alshraifeen et al. (2020) [21]	202	Hemodialysis patients exhibit a moderate degree of optimism. Researchers found low mean scores of individuals' quality of life and observed restricted motion among them. However, researchers indicate high success levels in the social interaction and psychological domains. A higher degree of hope was associated with a higher standard of living.
Mirbagher-Ajorpaz et al. (2016) [22]	300	The findings revealed a substantial and inverse correlation between hope, anxiety, and depression. Meanwhile, the duration of dialysis, anxiety, and depression also show a significant inverse relationship.
Yucens et al. (2019) [23]	65	Patients with chronic kidney disease (CKD) exhibited elevated levels of depression, decreased hope, and an increased prevalence of non-functional coping strategies in comparison to the healthy control group. The findings revealed that anxiety and depression negatively impacted the hopes of patients with CKD, whereas family social support positively impacted patients' hopes.
Poorgholami et al. (2016) [24]	50	Hope increased significantly in the intervention group (trained) as a result of stress management training in comparison to the control group (untrained).
Tabiban et al. (2017) [25]	120	Hope was significantly affected by an illness perception-based intervention for hemodialysis patients.
Sabouri et al. (2023) [26]	80	The intervention group exhibited substantially higher mean scores on hope and adherence to treatment following the positive thinking training intervention, as compared to the control group.

Effect of hope therapy on depression, anxiety, and stress of hemodialysis patients

Kidney therapies are necessary for those with end-stage renal failure to ensure their survival. Hemodialysis is a commonly used treatment method [27]. Around one million people worldwide receive hemodialysis, according to the currently available data [28]. In particular, 18,000 hemodialysis patients were estimated in 2007 [29,30], and by 2012, that number had risen to 20,000 in Iran [31]. Over the past thirty years, there has been a growing number of studies examining the impact of psychosocial factors on individuals with end-stage chronic renal failure [32,33]. Hemodialysis frequently results in patients' incapacity and restricts their daily activities, causing significant mental strain, worry, and sadness [34]. These difficulties lead to a reduction in the quality of life, increased hospitalizations, elevated healthcare expenses, and premature death [35]. Mollahadi et al. [36] conducted a study in Tehran to assess the levels of stress, anxiety, and depression in hemodialysis patients and kidney transplant (KT) patients. The study revealed that 51.7% of patients in the hemodialysis group and 38.4% in the KT group experienced stress. This study shows a notable prevalence of stress among individuals undergoing hemodialysis and kidney transplant (KT), with a higher occurrence observed among hemodialysis patients [36]. The duration of treatment and inadequate support systems for patients contribute to their inability to handle stressful situations, resulting in increased anxiety. A significant proportion of patients with chronic diseases exhibited these outcomes, as their condition was the reason for high stress levels.

Numerous studies have reported on the prevalence of anxiety among hemodialysis patients, with reported rates ranging from 27% to 45.7% [37-39]. The World Health Organisation (WHO) has forecasted that depression will rank as the second leading cause of disability among non-communicable diseases by the year 2020 [40]. It ranks as the fourth leading cause of death globally [41]. Statistics indicate that a significant proportion of hemodialysis patients experience depression, with rates ranging from approximately 20% to 70% [39]. 

Depression is one of the most prevalent psychological disorders. Hemodialysis patients gradually suffer from more mental health issues as a result of changes in their lifestyle and a decline in their social functioning [42]. Hence, it is essential to carry out the necessary procedures to treat these patients. Hope therapy is one of the techniques used to provide psychotherapy for hemodialysis patients. Schneider et al. claim that hope is a cognitive process that includes establishing goals, pursuing them, coming up with plans for how to get there, increasing motivation, and keeping track of your progress [40]. Hemodialysis patients use a lot of medications, most of which affect their kidneys; thus, adding more medications to manage their mental health problems could lead to the development of new problems. 

In this review, we observed that Rahimipouri conducted research to give hope to hemodialysis patients in seven sessions [11]. The seven sessions began with an explanation of the treatment process by the researcher. The topic of discussion in the second session was developing hope and how it affects stress, anxiety, and depression. While the third session included problem-solving strategies and participant anecdotes, the fourth session focused on identifying and preventing the occurrence of hope. During the fifth session, participants listed their most important life experiences, evaluated their relevance, and ranked their satisfaction. The sixth session's key subjects were goal-setting and improving mental and physical comprehension to overcome challenges. The seventh session focused on analyzing appropriate routes and helping patients to find efficient strategies. The results of their research indicate a significant decrease in depression, anxiety, and stress levels following hope therapy, as compared to the scores before the intervention. Their research has demonstrated that hope is a protective factor against depression and anxiety, as well as a predictor of fewer recurrent episodes of these conditions. Researchers found that after treatment, hemodialysis patients have a realistic goal and reduce pessimistic sentiments, which boosts their confidence. As a result, when faced with difficult situations, individuals exhibit increased resilience. Conversely, this research concluded that hope is an emotion that amplifies the likelihood of favorable actions or occurrences in the future [11].

Positive thoughts are most effective when shared and transmitted among group members. Group therapy benefits patients in several ways, including the opportunity to build relationships with people, observe and learn from peers, and gain support from fellow group members [12]. Through group therapy, people become aware that their peers experience similar struggles, which lessens their hesitation to admit and deal with the condition [14]. Ahmadifajr demonstrated that hemodialysis patients exhibited notable prevalence rates of overt and covert anxiety disorders. Additionally, they displayed a moderate degree of functioning in terms of adhering to their treatment across the four dimensions. Their results revealed that group therapy intervention successfully reduced the patients' visible and hidden anxiety while also improving their adherence to the treatment in various aspects. Their study concluded that patients who participated in the group hope therapy program acquired the skills necessary to create goals that are quantifiable, visible, and reachable. They also learned to modify these goals following their unique needs and capabilities [13]. 

Omranifard and colleagues [17] indicated that the severity of depression influences the level of adherence to treatment in hemodialysis patients. Therefore, it is crucial to prioritize and address the treatment of depression with increased attention and focus. Furthermore, Mirbagher-Ajorpaz et al. (2016) found that there was an inverse and significant correlation between anxiety and depression with the "hope" variable. Also, the results of their study showed that, similarly, there was an inverse and significant relationship between anxiety and depression with the duration of dialysis [22]. Yucens et al. (2019) revealed that the group of patients with chronic kidney disease (CKD) had lower levels of hope, higher levels of depression, and more dysfunctional coping strategies compared to healthy people. The results showed that anxiety and depression variables have adverse effects on the hope of patients with CKD. In contrast, social support from families has a positive impact on the hope of patients [23].

Hope and spirituality of hemodialysis patients

The spiritual condition of individuals undergoing hemodialysis and chronic kidney disease has a profound impact on their capacity to cope and is a dynamic component of the entire process. Ottaviani [14] conducted a study investigating the degrees of hope and spirituality in patients diagnosed with chronic renal disease undergoing hemodialysis treatment, showing a moderate positive correlation among variables. Gerogianni and colleagues [12] have shown that elements such as social support, spirituality and religiosity, remote nursing programs, and participation in support groups contribute to increased hope and reduced symptoms of anxiety and depression in individuals undergoing hemodialysis. Furthermore, Cha and colleagues have asserted that the connection between spirituality and hope depends on various factors, including affluence, occupation, and religious beliefs. Afazel's [18] research concluded that spiritual therapy can favor the level of hope in patients undergoing hemodialysis. Therefore, given the unique challenges faced by patients undergoing hemodialysis and the potential impact of depression on their overall welfare, it is highly recommended to integrate spiritual counseling into their treatment protocols. This type of counseling is economically efficient and has the potential to significantly impact the patient's physical, spiritual, and mental well-being favorably. Healthcare authorities, administrators, and scholars must prioritize the implementation of spiritual counseling and give it the necessary attention. 

Discussion

Previous research has demonstrated that the hope therapy program has a positive and statistically significant impact on the recovery of various diseases; therefore, combining this treatment method with pharmaceutical therapy may facilitate a more rapid and effective recovery from multiple diseases. Casellas-Grau et al. (2014) conducted a review that revealed that hope therapy in patients with breast cancer promotes the development of concentration, positive thoughts and relationships, strengths, interaction, success, life satisfaction, well-being, optimism, growth, and character formation [43]. Ilaghi et al. (2017) found that implementing hope therapy significantly and positively impacts the satisfaction levels of type 2 diabetes patients [44].

Hemodialysis patients may experience feelings of worry, stress, and sadness during treatment, which could make it difficult for them to follow recommended treatment plans. As a result, the use of non-therapeutic techniques like hope therapy can improve intervention adherence to treatment and reduce anxiety, tension, and depression among them. Rahimipour et al. (2015) revealed that hemodialysis patients' mean levels of anxiety, stress, and sadness were significantly lower after inducing hope therapy in treatment [11]. Georgian et al. (2019) found that participation in support groups, spirituality and religion, social support, and remote nursing programs all help hemodialysis patients experience and reduce their feelings of depression and anxiety [12]. 

Ahmadifajr et al. (2023) observed that group therapy had a significant impact on reducing anxiety and increasing treatment adherence among hemodialysis patients [13]. On the other hand, Farnia et al. (2016) revealed that hemodialysis patients experienced a substantial increase in their overall happiness score after implementing the hope therapy intervention [16]. The research conducted by Mirbagher-Ajorpaz et al. (2016) revealed a statistically substantial inverse correlation between the variable "hope" and both anxiety and depression. Furthermore, their findings indicated a noteworthy and inverse correlation between the duration of dialysis and both anxiety and depression [22]. Yucens et al. (2019) observed that individuals diagnosed with chronic kidney disease (CKD) exhibited low hope, elevated depressive symptoms, and a greater propensity for employing dysfunctional coping mechanisms in comparison to the healthy control group. The findings revealed that anxiety and depression negatively impact the hope of patients with chronic kidney disease (CKD), whereas familial social support positively influences patients' hope [23].

Maintaining hope and reducing anxiety disorders are considered crucial for hemodialysis patients, as supported by the majority of prior research [18,19,24-26]. Research has employed a variety of methodologies to reduce anxiety and increase hope among dialysis patients. Afazel et al. (2013) observed that spiritual counseling significantly influences the levels of hope among hemodialysis patients [18]. Furthermore, similar findings were observed in the Oshvandi et al. study, in which they revealed that spiritual care positively impacted the motivation and hope levels of hemodialysis patients [19]. Poorgholami et al. (2016) observed that stress management training had a substantial positive impact on the hope of the intervention group (trained) when compared to the hope of the control group (non-trained) [24]. Tabiban et al. (2017) also observed a significant influence of disease perception-based interventions on the optimism of hemodialysis patients [25]. Likewise, Sabouri et al. (2023) reported that following the positive thinking training intervention, the intervention group exhibited a significantly higher mean score on hope and treatment adherence (26 points) than the control group. Maintaining consistency in implementing positive psychology programs is essential for the hope treatment approach to facilitate the recovery of various disorders, notwithstanding their positive effects. 

## Conclusions

Based on the findings of similar studies conducted in the past, it is possible to conclude that hope therapy can increase hemodialysis patients' happiness, quality of life, and treatment adherence while decreasing anxiety, tension, and depression. In addition, effective interventions for enhancing the hope of hemodialysis patients include spiritual counseling, spiritual therapy, stress management training, interventions based on disease perception, positive thinking training, and similar approaches. While it has been proven that hope therapy and other positive interventions can assist in the faster and more favorable recovery of hemodialysis patients, these therapies must be consistently employed to ensure optimal patient results.
